# Co-Design Method and Wafer-Level Packaging Technique of Thin-Film Flexible Antenna and Silicon CMOS Rectifier Chips for Wireless-Powered Neural Interface Systems

**DOI:** 10.3390/s151229885

**Published:** 2015-12-16

**Authors:** Kenji Okabe, Horagodage Prabhath Jeewan, Shota Yamagiwa, Takeshi Kawano, Makoto Ishida, Ippei Akita

**Affiliations:** 1 Department of Electrical and Electronic Information Engineering, Toyohashi University of Technology, Aichi 441-8580, Japan; okabe-k@int.ee.tut.ac.jp (K.O.); jeewan@int.ee.tut.ac.jp (H.P.J.); yamagiwa-s@int.ee.tut.ac.jp (S.Y.); kawano@ee.tut.ac.jp (T.K.); ishida@ee.tut.ac.jp (M.I.); 2 Electronics-Inspired Interdisciplinary Research Institute (EIIRIS), Toyohashi University of Technology, Aichi 441-8580, Japan

**Keywords:** wireless power transmission, wafer-level packaging, flip-chip bonding, flexible substrate, rectenna

## Abstract

In this paper, a co-design method and a wafer-level packaging technique of a flexible antenna and a CMOS rectifier chip for use in a small-sized implantable system on the brain surface are proposed. The proposed co-design method optimizes the system architecture, and can help avoid the use of external matching components, resulting in the realization of a small-size system. In addition, the technique employed to assemble a silicon large-scale integration (LSI) chip on the very thin parylene film (5 μm) enables the integration of the rectifier circuits and the flexible antenna (rectenna). In the demonstration of wireless power transmission (WPT), the fabricated flexible rectenna achieved a maximum efficiency of 0.497% with a distance of 3 cm between antennas. In addition, WPT with radio waves allows a misalignment of 185% against antenna size, implying that the misalignment has a less effect on the WPT characteristics compared with electromagnetic induction.

## 1. Introduction

Advances in techniques employed in wireless sensor systems have enabled the creation of novel biomedical applications [1,2]. In particular, neural interface systems including micro-electrode arrays and signal processing circuits have been studied to identify human brain functions based on the weak electrical signals caused by the activity of nerve cells in the brain [3,4,5,6,7,8,9,10]. The signals obtained from the neural interface are essential for realizing brain-machine interfaces and supporting the lack of information in the brain caused by disorders and diseases. However, neural recording systems that use wire lines to connect the implanted device to an external device can cause infections through the opening in the skull and the dura. Although the skull is typically be sealed with cement after surgery, it would be difficult to hold the wire and the dura. As a result, there is a risk of infection and leakage of the cerebrospinal fluid during long-term measurement. Therefore, using fully implantable neural interfaces are necessary to solve this problem [11,12,13,14,15,16,17].

For realizing wireless communication and power transmission to the implanted neural interface on the brain surface, several technologies that integrate passive components (e.g., an antenna on a flexible film) with high-performance active circuits have been studied [18,19,20,21,22]. In particular, many functional circuits including amplifiers, analog-to-digital converters, signal processors, and RF circuits are needed and they should all support low-power operation [23,24,25,26,27,28,29,30,31,32,33]. Very thin film flexible transistors fabricated with organic or bio-resorbable materials can be used to fabricate monolithic film devices with passive and active components. However, these flexible transistors require a large area for the multi-functional circuits because the gate size of the transistors exceeds 15 μm caused by evaporation through a shadow mask [18]. Therefore, implementing highly integrated systems using flexible transistors might be difficult. As an alternative, fabrication processes that integrate a complementary metal-oxide semiconductor (CMOS) IC on a flexible substrate have been developed [19,20,21,22,23]. Although the mounting method based on a micro electro-mechanical system (MEMS) technology enables accurate alignment in the integration of the CMOS IC chip and the flexible film device, this alignment becomes challenging when mounting several chips on the same film [19,20]. To overcome this challenge, a mounting technique using the flip-chip bonding method has been studied. This method can help achieve good alignment even if several chips are packaged on a flexible substrate [21,22]. A polyimide film and a CMOS IC chips have also been integrated using flip-chip bonding technology [21]. Because this polyimide film has a certain thickness, the fabricated device exhibited sufficient flexibility and hardness, making it suitable to be implanted on the surface of the eyeball. However, the neural interface implanted on the brain surface requires that the flexible film be thinner in order to fit the shape of the brain [6]. Therefore, there is a need for a technique that can be used for mounting CMOS IC chips on a very thin film device.

The design methodology of RF circuits is also important to realize such small-size implantable devices. In the wireless power transmission (WPT) device, an inductance element is generally required for matching the impedance between an antenna and a rectifier [23], that is, external components are required. Considering the size constraint of the implantable device, a design method that eliminates such matching components should be considered. Although [24] achieves a small inductance for matching impedance by using an inductive antenna, it is difficult to apply in implantable devices because this antenna is relatively large and thick, 50 mm × 43 mm × 0.5 mm. Therefore, an antenna and a rectifier (rectenna) co-design method must be considered to reduce the use of matching components and miniaturize the antenna size. 

In this paper, we propose a co-design method and an assembly technique of a flexible antenna and the CMOS rectifier chip for realizing small-sized implementation of implantable neural interfaces. The advantages of the proposed technique are that it does not require any off-chip matching components between the antenna and the rectifier, and it allows integration of a high-performance silicon chip on an ultra-thin flexible film with a thickness of 10 μm. For minimizing the size of the implantable device, the antenna is designed to have a small inductance to avoid using additional matching components between the antenna and the rectifier. Furthermore, an on-chip transformer preceding the rectifier can reduce the inductance, resulting in a smaller inductive antenna device. In addition, we have developed a wafer-level packaging technique for mounting the CMOS IC chip on a thin film with flip-chip bonding. To the best of our knowledge, an assembly technique on a thin film with a thickness of 5 μm has never been reported in the past. Although a previous study [34] by the authors reported a silicon CMOS rectifier chip mounted on the antenna with the flexible substrate and demonstrated WPT, detailed design methodologies and discussions were not provided. This paper presents additional information on an impedance matching method and analysis, the flip-chip bonding process, and additional measurement results and discussion for realizing small-sized devices.

The remainder of this paper is organized as follows: in Section 2, we present the architecture and the co-design method of the flexible rectenna that contains a flexible antenna, an on-chip transformer, and a CMOS rectifier for impedance matching. Section 3 explains the fabrication process for the flexible device, especially the flip-chip bonding technology to connect a silicon chip. In Section 4, we demonstrate WPT by the fabricated device, and discuss the power transfer efficiency by comparing the measured and calculated results. Finally, conclusions are provided in Section 5.

## 2. Flexible Rectenna Device

### 2.1. Architecture of the Implantable Device

**Figure 1:**
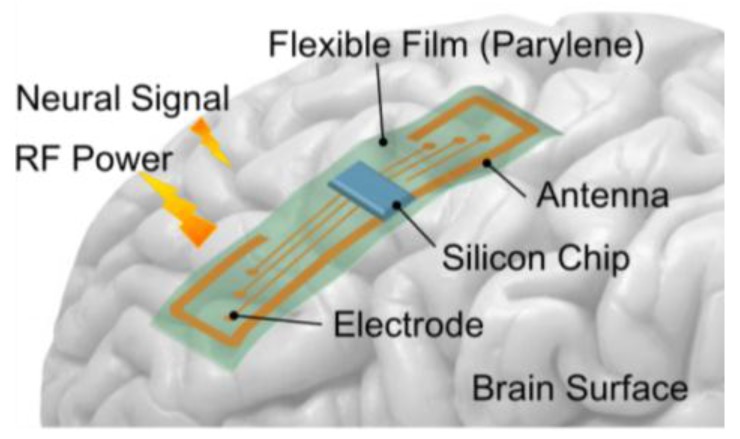
Proposed architecture of the flexible device for wireless-powered neural interface systems.

The proposed architecture of the flexible device implanted on the brain surface is shown in Figure 1. Highly functional active circuits are implemented in a silicon chip that is embedded in a flexible film. Some passive components, including the antenna and the electrode array, are also patterned in the flexible film. A thin parylene film is selected as the flexible material because of its good flexibility, which allows it to fit the brain surface, and its high biocompatible. In this paper, as a WPT part is considered, a co-design methodology of circuits and the antenna is needed for achieving the optimum design in terms of device size and performance, and the methodology is described in the next section.

### 2.2. Co-Design Methodology

**Figure 2:**
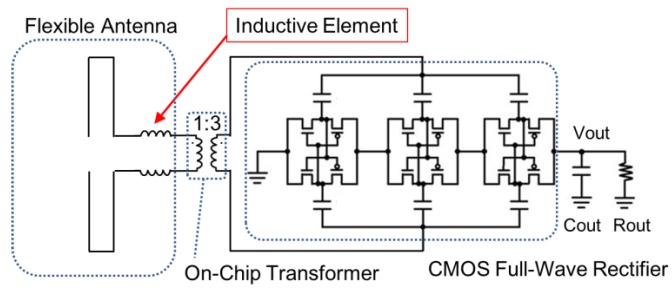
Circuit schematic of the proposed WPT device: The antenna with inductance can help eliminate the need for using external matching components, thus facilitating a reduction in the size of the neural interface device.

Figure 2 shows the circuit diagram of the flexible rectenna designed for WPT. The flexible rectenna has three parts: a flexible antenna having a small inductance, an on-chip transformer for matching the impedance between the antenna and the rectifier, and a rectifier circuit. In this schematic, the antenna is made of metal in the parylene film and the other parts are implemented in a 180-nm standard CMOS process. The rectifier circuit is formed by cascading in three stages and it has charge pump capability for DC–DC conversion [35]. To design the on-chip transformer between a 50-Ω terminated antenna and the CMOS rectifier, the input impedance of the rectifier should be calculated assuming that the MOS transistor is switched on. The received antenna would be able to receive about 700 μW (−1.54 dBm), which is determined by available received power on the human brain [17]. Therefore, the gate size of MOS transistors in the rectifier and the on-chip transformer were designed to match the impedance of rectifier and antenna when the received power is 700 μW. The ratios of the gate width to the gate length (*W/L*) for n-MOS and p-MOS transistors are 66.6 and 200, respectively, and the threshold voltage (*V_TH_*) is approximately 350 mV. The CMOS rectifier is activated when a voltage greater than 350 mV is applied to the RF terminals that are connected to the antenna elements. The on-resistance of the MOS transistor (*R_ON_*) is calculated as:

(1) $${\text{R}}_{\text{ON}}=\frac{1}{\beta \left({\text{V}}_{\text{GS}}-{\text{V}}_{\text{TH}}\right)}$$

where *β* is the parameter decided by the *W/L* ratio, the carrier mobility, and the unit capacity of gate, and *V_GS_* is the gate-to-source voltage. Equation (1) indicates that *R_ON_* depends on the input power because *V_GS_* is determined by the transmitted power from the antenna to the CMOS rectifier. Therefore, a certain level of input power range into the rectifier should be defined in order to specify *R_ON_*. In the case of the designed CMOS rectifier circuit, the real part of the input impedance varies from 100 Ω to 650 Ω, when the input power is sufficiently high. Consequently, the on-chip transformer should be designed to have a turn ratio of 1:3, implying that the real-part input impedance of antenna is seen as 450 Ω from the rectifier side. The on-chip transformer with silicon substrate is designed with a turns ratio of 2:6 and size of 800 μm × 800 μm with an electromagnetic simulator (Momentum, Keysight Technologies, Santa Rosa, CA, USA). The width and space of the coil are 4 μm and 3 μm, respectively, and the self-resonant frequency is higher than 3 GHz in this design. The transformer has maximum efficiency at a frequency of 825 MHz when the outer diameter of the transformer is adjusted to 800 μm. The transfer characteristic of the designed transformer is approximately −3.4 dB at the frequency of 825 MHz. In addition, some inductance components are required for matching the impedance between the antenna and the rectifier because the rectifier has capacitive impedance. This unintended negative reactance can be cancelled using an inductance of 3.4 nH, which can be easily added in the antenna design. Thus, the flexible antenna having a small inductance can help eliminate the need for using extra inductance components.

**Figure 3:**
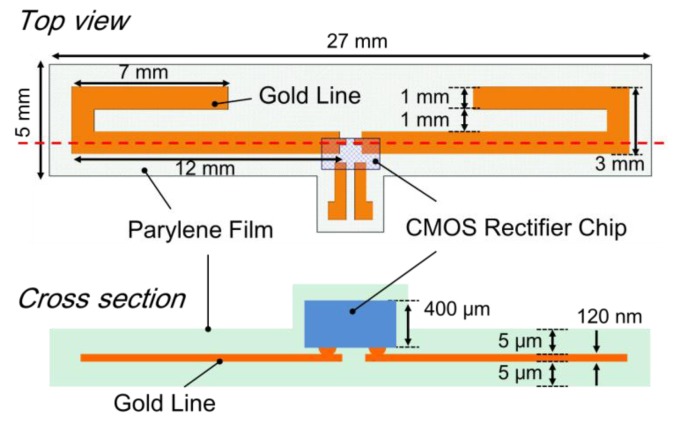
Diagram of the designed flexible antenna with the CMOS rectifier chip.

**Figure 4:**
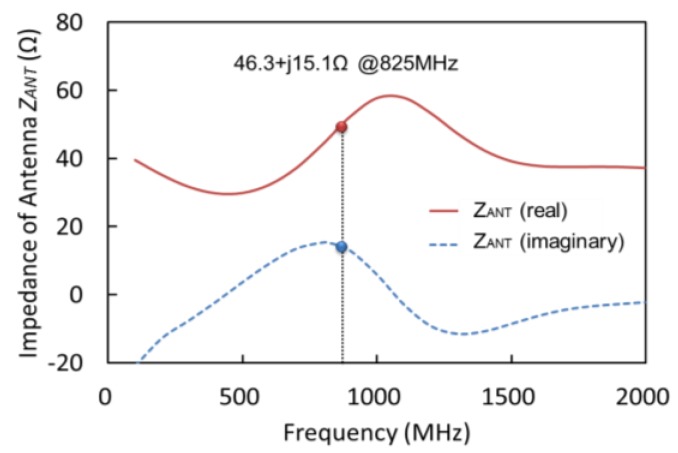
Simulated input impedance of the designed flexible antenna in a saline model.

**Figure 5:**
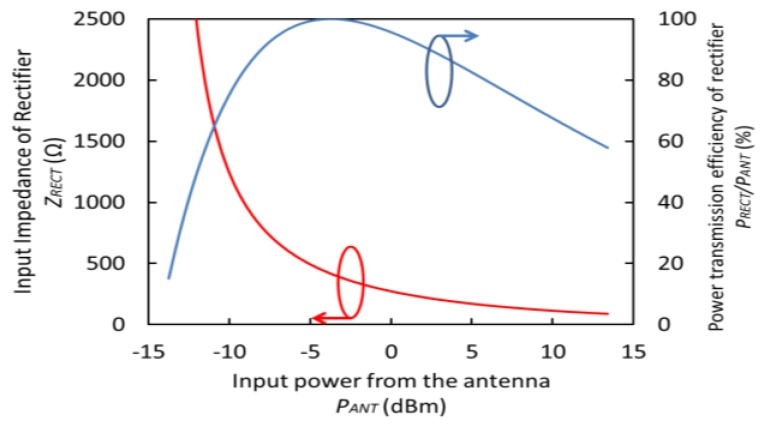
Input impedance (*R_RECT_*) and power transmission efficiency (*P_RECT_/P_ANT_*) of the rectifier as a function of input power (*P_ANT_*).

The structure of the flexible and inductive antenna is based on a dipole antenna model and the antenna is designed using an electromagnetic simulation engine (EMPro, Keysight Technologies). The model of dipole antenna was adopted in order to form on the flexible film in plane. The antenna was designed on the flexible film with a large area of 27 mm × 5 mm, as the gain of the antenna depends directly on its size. The flexible antenna is analyzed in a saline model that represents the brain tissue. Figure 3 shows the diagram of the designed flexible antenna with the CMOS rectifier chip. The antenna’s metal line is patterned using gold, with a width of 1 mm and thickness of 120 nm. The flexible film has a size of 27 mm × 5 mm × 10 μm, and the assembled silicon chip has a thickness of 400 μm. 97% of the device area is composed of a flexible film, as the silicon chip has a small area of 1.5 mm × 2.5 mm only. The simulated input impedance of the antenna is shown in Figure 4 and it indicates an impedance of 46.3 + j15.1 Ω at 825 MHz, indicating that the designed antenna has an inductance of 3 nH. From these simulation results, the power ratio, defined as the input power into the rectifier (*P_RECT_*) divided by the transmitted power from the antenna (*P_ANT_*), is estimated as:

(2) $$\frac{P_{RECT}}{P_{ANT}}=\frac{4\cdot Z_{ANT}\cdot Z_{RECT}}{{\left(Z_{ANT}+Z_{RECT}\right)}^{2}}$$

where *Z_ANT_* and *Z_RECT_* are the input impedances of the antenna and the rectifier, respectively. *Z_RECT_* depends on the gate voltage as described previously. Figure 5 shows the input impedance of the rectifier (*Z_RECT_*) and the efficiency of power transmission to the rectifier from the antenna (*P_RECT_/P_ANT_*) *versus* the input power from the antenna (*P_ANT_*). The power loss due to impedance mismatch is less than −1.0 dB in the range of the input power from −10 to 5 dBm.

## 3. Integration of Flexible Film and Silicon Chip

**Figure 6:**
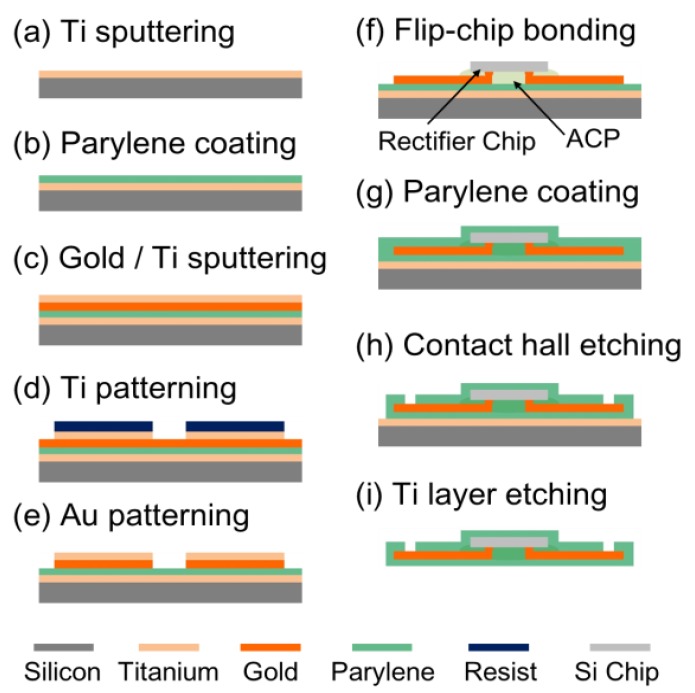
Fabrication process of flexible rectenna mounting the CMOS rectifier chip with the transformer on the parylene film.

**Figure 7:**
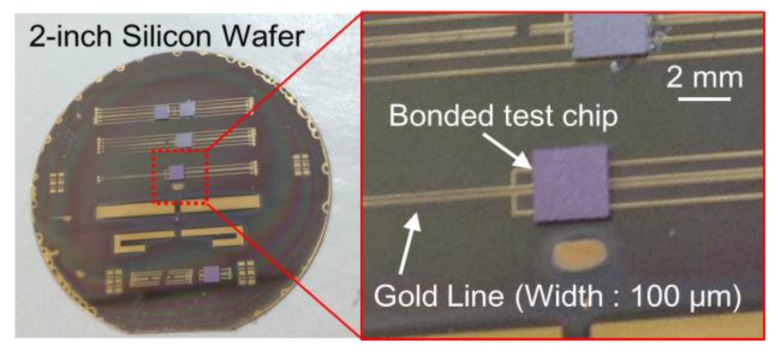
Photograph after flip-chip bonding process (Figure 6f).

The flexible rectenna was fabricated and assembled by using a 2-inch silicon wafer and the fabrication process illustrated in Figure 6. The parylene layer device was fabricated using reported processes [6,9]. A 40-nm thick titanium layer was sputtered on the silicon wafer as a sacrificial layer (Figure 6a). Then, a parylene layer of thickness of 5 μm was deposited on the titanium layer (Figure 6b), and a 120-nm gold layer was sputtered as a metal line on the parylene film. In addition, a titanium layer was sputtered again on the gold layer as a metal mask to pattern the gold layer later (Figure 6c). The top titanium layer was etched for the antenna pattern by photolithography (Figure 6d), and then, the resist was removed. After the photolithography process, the gold layer was etched by aqua regia at room temperature (Figure 6e). After removing the top titanium layer, the silicon chip was connected to the antenna pads by flip-chip bonding with an anisotropic conductive paste (ACP). An ACP named TAP0402E (Kyocera, Kyoto, Japan) containing nickel particles with a diameter of 2 μm was used. In this process, the bonding conditions of pressure, time, and temperature were decided using a test chip because the thin parylene film would break if the selected conditions were not appropriate. The test chip has the size of 2.5 mm × 2.5 mm, and the four electrode pads of size 100 μm × 100 μm each are separated by distance of 100 μm. Gold bumps were formed on each pad of the test chip, and the chip was connected to the gold line formed on the parylene film. In order to form firm bonding using the ACP, the connected device was kept at 100 °C for 3 min. Figure 7 is a photograph taken after flip-chip bonding using the test chip, and the chip was firmly jointed to the parylene film. The misalignment between the gold line and the chip pads became less than 2 μm owing to the flip-chip bonding technology employed. In this wafer-level packaging technology, several chips can be assembled high accuracy. In addition, good electrical characteristics were obtained during conductive (<30 Ω) and insulation (>100 MΩ) measurements. After bonding the CMOS rectifier chip (Figure 6f), a parylene layer of thickness 5 μm was deposited (Figure 6g). The output pads were opened using the titanium mask by plasma etching, and then, the parylene film was patterned (Figure 6h). Finally, by etching the sacrificial titanium layer, the parylene film with the chip was released from the silicon wafer (Figure 6i).

**Figure 8:**
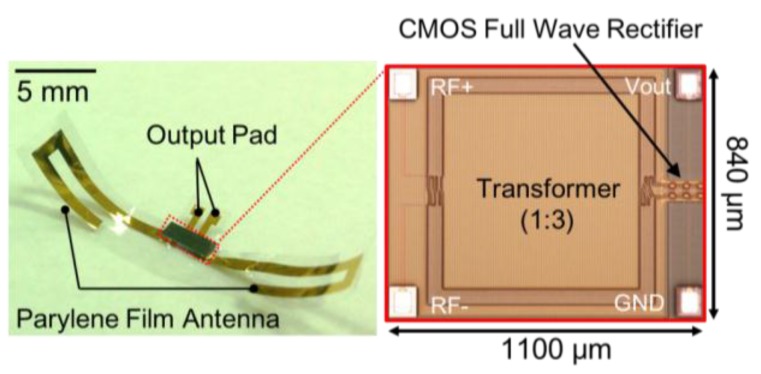
Photographs of the fabricated flexible rectenna and the bonded CMOS rectifier chip with the transformer.

Figure 8 shows the fabricated parylene film device and the bonded on-chip full wave CMOS rectifier with the transformer. The size of the flexible device is 27 mm × 5 mm and it has a good flexibility. The CMOS rectifier chip of size 1100 μm × 840 μm has four pads for providing rectified output voltage, GND, and the RF signal terminals.

## 4. Measured Results and Discussion

**Figure 9:**
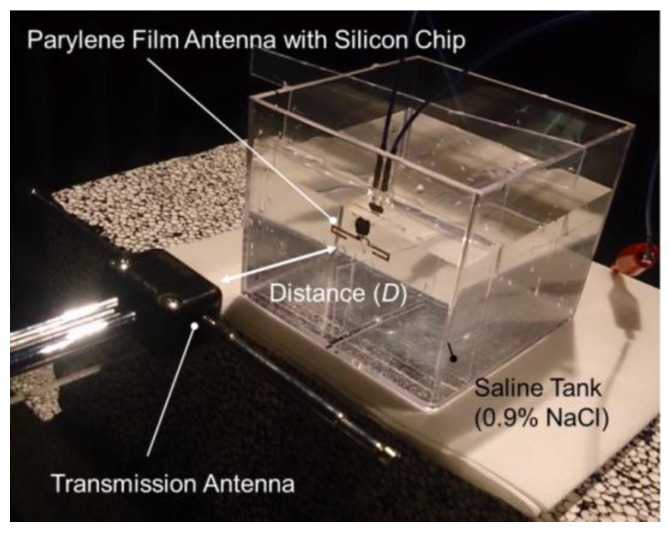
Measurement setup for WPT demonstration using saline tank in anechoic chamber.

**Figure 10:**
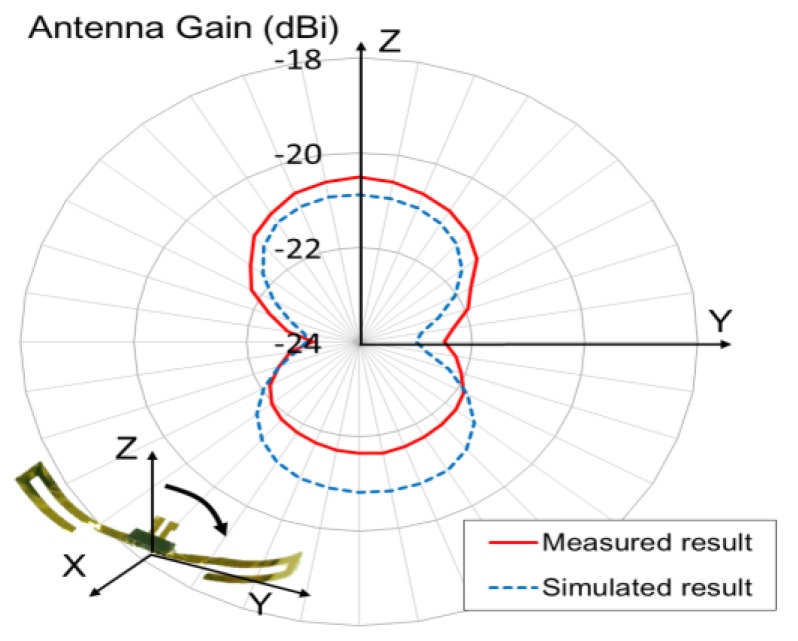
Radiation characteristics of fabricated flexible antenna immersed in saline tank.

As the fabricated rectenna device is designed for use on the brain surface, the performance of the device was measured in a saline model that served as the emulation of the implant [19]. Figure 9 shows the experimental setup with the saline tank (0.9% NaCl solution) in an anechoic chamber. First, an antenna device without a rectifier was evaluated to characterize the flexible antenna, and its input impedance of 41.2 + j44.3 Ω was observed at the resonance frequency of 825 MHz. In this case, the measured return loss was −7.02 dB. The fabricated antenna had a gain of −20.5 dBi in the direction of Z-axis, a value that matches well with the simulated result of −20.9 dBi as shown in Figure 10. Therefore, the simulation can estimate the reasonable antenna gain as it includes the influence of a saline environment, thus facilitating the measurement of the total efficiency of the WPT system. For example, the free-space loss is 10.8 dB at a frequency of 825 MHz and at a distance of 10 cm between each antenna. Therefore, the WPT efficiency between each antenna can be estimated to be −29.6 dB, if the gains of the transmission dipole antenna and the receiver antenna are 2.14 dBi and −20.9 dBi respectively.

**Figure 11:**
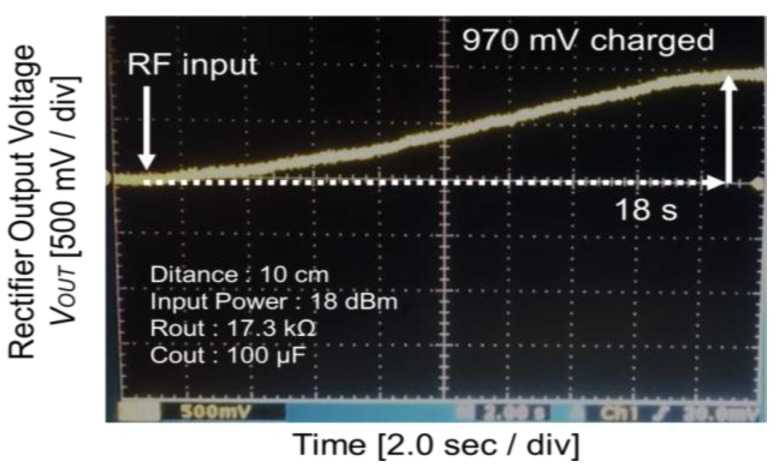
Measured output voltage waveform of the rectifier in WPT.

We demonstrated WPT using the flexible rectenna that integrates the flexible antenna and the CMOS rectifier chip with the transformer. The RF power at 825 MHz was transmitted using a standard half-wavelength dipole antenna at a distance of *D* from the flexible antenna. The load resistance *R_OUT_* and the load capacitance *C_OUT_* were connected to the output port of the rectifier. Figure 11 shows the observed rectifier output voltage waveform when *D* = 10 cm, the input power was 18 dBm, and the load resistance and load capacitance were 17.3 kΩ and 100 μF, respectively. The output capacitor was charged up when the RF signal was input and it was confirmed that the output voltage increased to 970 mV after 18 s.

**Figure 12:**
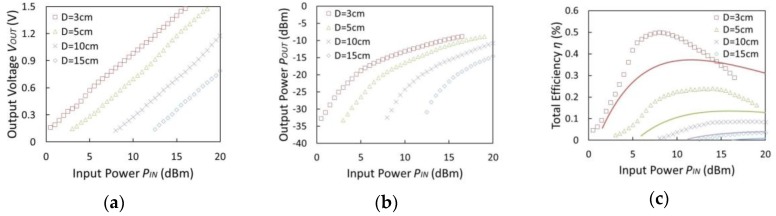
Measured output voltage (*V_OUT_*), output power (*P_OUT_*), and total efficiency (*η*) *versus* input power (*P_IN_*) for different distances (*D*). (**a**) *V_OUT_* *versus* input power; (**b**) *P_OUT_ versus* input power; (**c**) *η* *versus* input power.

**Figure 13:**
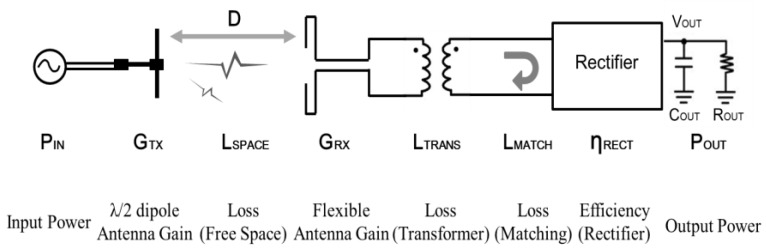
Block diagram of the WPT system.

Figure 12a–c show the output voltage, the output power, and the total efficiency *versus* the input power of the transmission antenna at distances 3, 5, 10, and 15 cm, respectively. The output resistance was adjusted to maximize the total efficiency such that the output voltage did not exceed 1.5 V, as shown in Figure 12a. The output voltage and the output power decreased as the distance between the antennas becomes longer. In these measurement results, the maximum efficiency was 0.497% at a distance of 3 cm and input power of 8 dBm, and the efficiency obtained compares well with the calculated value of 0.36%. The solid lines in Figure 12c depict the theoretical efficiencies which are calculated using the block diagram of the WPT system shown in Figure 13. The total efficiency, *η* (%), is calculated as:

(3) $$\eta =\frac{P_{OUT}}{P_{IN}}\times 100=G_{TX}\cdot L_{SPACE}\cdot G_{RX}\cdot L_{TRANS}\cdot L_{MATCH}\cdot \eta_{RECT}$$

where *P_IN_* and *P_OUT_* are the input power of the transmission antenna and the output power consumed in load resistor *R_OUT_*, respectively. *G_TX_* and *G_RX_* are antenna gains of transmission and reception, *L_SPACE_* is the free space loss, *L_TRANS_* is the transformer loss, *L_MATCH_* is the mismatch loss calculated by Equation (2), and *η_RECT_* is the efficiency of the CMOS rectifier. The free-space loss can be calculated by the Friis transmission equation as:

(4) $$L_{SPACE}=10\cdot \log_{10}{\left(\frac{\lambda }{4\pi D}\right)}^{2}$$

where *D* is the distance and *λ* is the wave length. The calculated power efficiency from Equation (3) has similar curves compared with measured values as shown in Figure 12c. The peak of total efficiency characteristics depends on the input power into the flexible antenna (*P_ANT_*), which is determined by *P_IN_*, *G_TX_*, *G_RX_*, and *L_SPACE_*. Therefore, the characteristic of the total efficiency is affected by the characteristic of *L_MATCH_* determined by *P_IN_* and distance between each antenna.

**Figure 14:**
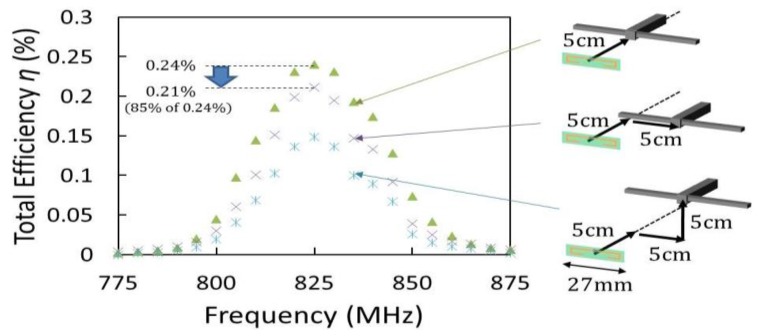
Frequency response of total efficiency (*η*) for different positions (*D* = 5 cm).

The frequency characteristics of the total efficiency for different positions of the transmission antenna are shown in Figure 14. The peak efficiency was observed at the resonance frequency of 825 MHz. Figure 14 also shows the characteristics of efficiency regarding a misalignment in horizontal and vertical direction when the distance is 5 cm between antennas and there is not misalignment in angle between antennas. The transmission antenna was moved by 5 cm in horizontal and vertical direction. As a result, the efficiency became 0.21% from 0.24% when the misalignment in horizontal direction is 5 cm, which is 185% of the fabricated antenna size (27 mm). Comparing with WPTs using electromagnetic induction, the misalignment of the implanted coil and the outer devices drastically affects the efficiency. The misalignment of 10% against coil size causes the WPT efficiency to decrease to 85% in electromagnetic-induction-based WPTs [36]. By contrast, the fabricated RF-based device allows the misalignment of 185% against antenna for the same efficiency reduction of 85%. Therefore, the fabricated device has a tolerance to misalignment between the implanted and outer devices in terms of WPT efficiency loss. Table 1 shows the comparison results of the WPT devices [37,38]. Although the method by radio waves has a lower power efficiency compared to electromagnetic induction, it has the advantages of long distance WPT, and it also allows for some misalignment between the implanted and outer devices. As seen from Figure 12c, the fabricated rectifier outputs the power (*P_OUT_*) of 22 μW when *D* = 5 cm and *P_IN_* = 10 dBm. If the misalignment in horizontal direction is 5 cm, the output power (*P_OUT_*) becomes 19 μW, the value of which is 85% of the power without misalignment. This obtained power could drive a neural interface LSI, such as [37] by charging in a battery in temporarily. For instance, a battery can be charged with a constant current of several tens of micro amperes (μA) [39]. Since the loaded current in the implemented system can be calculated as 27 μA at the condition of *P_IN_* = 10 dBm and *V_OUT_* = 700 mV at *D* = 5 cm, the received power is reasonable to charge a battery.

**Table 1:** Comparison of WPT system with existing studies.

Study	Method	Device Size (Area, Thickness)	Transmission Efficiency (Tx to Rx)	Operating Frequency	Communication Distance
[37]	Electromagnetic induction	6.5 × 6.5 mm^2^, >5 μm	−17.3 dB	300 MHz	1.6 cm
[38]	Electromagnetic induction	5 × 5 mm^2^, >20 μm	−26 dB	118 MHz	4 cm
This work	Radio wave	5 × 27 mm^2^, 10 μm	−29.6 dB	825 MHz	10 cm

## 5. Conclusions

The co-design and assembly methods of the flexible antenna and the CMOS rectifier chip are proposed for fabricating implantable neural interfaces. The power loss from the flexible antenna into the CMOS rectifier is saved by using a well-designed on-chip transformer for the input power ranging from −10 to 5 dBm. The presented design method also eliminates the required inductance for impedance matching, resulting in less components and smaller sizes. The integrated flexible rectenna device with differential substrates has been fabricated with the flip-chip bonding technique to mount the silicon chip on the 5-μm thick parylene film. We have achieved to match the impedance of the antenna and the rectifier by using the flexible antenna with a size of 27 mm × 5 mm × 10 μm, and on-chip transformer with an area of 800 μm × 800 μm. The measured maximum efficiency was 0.497% with 3 cm between each antenna. Furthermore, the RF-based WPT allows the misalignment of 185% against the antenna size while maintaining more than 85% efficiency degradation, indicating the tolerability against a misalignment compared with electromagnetic induction.
